# STIP is a critical nuclear scaffolding protein linking USP7 to p53-Mdm2 pathway regulation

**DOI:** 10.18632/oncotarget.5303

**Published:** 2015-10-10

**Authors:** Mao Ye, Yani Tang, Shijun Tang, Jing Liu, Kuangpei Wu, Shan Yao, Yang Sun, Lei Zhou, Tanggang Deng, Ying Chen, Chenghan Huang, Weihong Tan

**Affiliations:** ^1^ Molecular Science and Biomedicine Laboratory, State Key Laboratory for Chemo/Biosensing and Chemometrics, College of Biology, College of Chemistry and Chemical Engineering, Collaborative Innovation Center for Molecular Engineering for Theranostics, Hunan University, Changsha, Hunan 410082, China; ^2^ School of Life Sciences, State Key Laboratory of Medical Genetics, Central South University, Changsha, Hunan 410078, China; ^3^ Department of Molecular Genetics and Microbiology & UF Shands Cancer Center, College of Medicine, University of Florida, Gainesville, FL 32610, USA; ^4^ Laboratory of Biochemistry and Molecular Genetics, New York Blood Center, New York, NY10065, USA

**Keywords:** USP7, deubiquitinating enzyme, Mdm2, p53, tenary protein complex

## Abstract

The ubiquitin-specific protease USP7 stabilizes both Mdm2 and p53 by removing ubiquitins, hence playing an important enzymatic role in the p53-Mdm2 pathway. However, it is poorly understood how USP7 executes its dual-stabilization effect on Mdm2 and p53 in cellular context. Here, we report that STIP is a novel macromolecular scaffold that links USP7 to the p53-Mdm2 pathway. STIP and a fraction of USP7 interact and constitutively colocalize in nucleoplasma. Overexpression of STIP stabilizes Mdm2 and p53, whereas downregulation of STIP decreases Mdm2 and p53 levels. The effect of STIP on Mdm2 and p53 depends on USP7 function as a deubiquitinating enzyme. Furthermore, we demonstrate that STIP mediates the assembly of two separate ternary protein complexes *in vivo* as STIP-USP7-Mdm2 and STIP-USP7-p53, which facilitates USP7-mediated stabilization of Mdm2 and p53. Collectively, these results pinpoint a new molecular function of STIP and reveal a novel mechanism whereby USP7 executes its dual-stabilization effect on Mdm2 and p53 via STIP scaffolding.

## INTRODUCTION

p53 is a multi-talented tumor suppressor, yet the most frequently mutated gene in human cancers [1, 2]. Extensive research has identified a complex network of p53 functions and revealed a central tenet at adapting its dynamic induction and activation [3, 4]. In untransformed cells, p53 is kept at low levels with a short half-life via ubiquitin-dependent proteasomal degradation [5, 6]. In response to stresses such as DNA damage, p53 is stabilized via decreased protein degradation and increased gene expression, resulting in cell cycle arrest, apoptosis, or cellular senescence [7]. Mdm2, an E3 ubiquitin ligase, is a key negative regulator of p53 that catalyzes its ubiquitination [8]. A cardinal feature of the mutuality between Mdm2 and p53 is their balancing act through reciprocal modulation. While under a positive feedback of p53, Mdm2 is itself subject to autoubiquitination [8, 9].

Studies have shown that p53 or Mdm2 ubiquitination is reversible before final commitment to proteasomal degradation. The deubiquitinase USP7 binds Mdm2 or p53 via its *N*-terminal TRAF-domain or C-terminal region in a mutually exclusive manner to remove ubiquitins and stabilize the two proteins [10–13]. The *in vitro* binding affinity of USP7 for Mdm2 is several-fold higher than for p53 [14, 15], implying that Mdm2 is likely the preferred substrate of USP7 in unstressed cells. In addition, USP7 may deubiquitinate p53 *in trans* through an Mdm2-mediated indirect interaction [16]. Thus, in absence of stress, USP7 mainly deubiquitinates and stabilizes Mdm2, while to a minor extent USP7 also deubiquitinates and stabilizes p53 [17]. However, upon DNA damage the affinity of USP7 for Mdm2 is reduced through ATM-dependent phosphorylation [10], tilting the balance toward p53 stabilization [16].

STIP (sip1/tuftelin interacting protein) belongs to a unique class of multidomain proteins that share a G-patch, a coiled-coil, and several short tryptophan-tryptophan repeats from the *N* to *C*-terminal region [18]. Proteomic studies identified STIP as a nuclear phosphoprotein in HeLa cells [19] and a factor potentially associated with the spliceosome [20, 21]. STIP self-aggregate into a rod-shaped, macromolecular structure called the stiposome in the nucleoplasm upon overexpression [18, 22]. In *C. elegans*, the knockdown of STIP by RNA interference (RNAi) led to complete arrest at the 16-cell stage in early embryos during development [18]. This lethal phenotype could be readily rescued with either a human or Drosophila STIP gene [18], pinpointing its essential roles in conserved biological processes. Nevertheless, the biochemical and cellular aspects of STIP function and mechanism have remained to be investigated.

We now report the identification of a novel molecular function of STIP that links USP7 to the p53-Mdm2 pathway. We found that STIP and USP7 interact and colocalize in the native nuclear compartment. Overexpression of STIP stabilized Mdm2 and p53, whereas downregulation of STIP decreased p53 and Mdm2 levels. We also provide evidence that STIP-mediated stabilization of p53 and Mdm2 depends on USP7 and show the existence *in vivo* of two ternary complexes as STIP-USP7-Mdm2 and STIP-USP7-p53. Given that STIP is not known to possess any enzymatic activity, we conclude that STIP functions as a nuclear scaffold that enables protein complex assembly and plays a critical role in the p53-Mdm2 axis by facilitating USP7-mediated stabilization of Mdm2 and p53. Collectively, these results pinpoint a new molecular function of STIP and reveal an important missing piece in the regulation of Mdm2 and p53 stability by USP7 in cellular context.

## RESULTS

### STIP interacts with USP7 and the two proteins colocalize to the nucleoplasma *in vivo*

**Figure 1:**
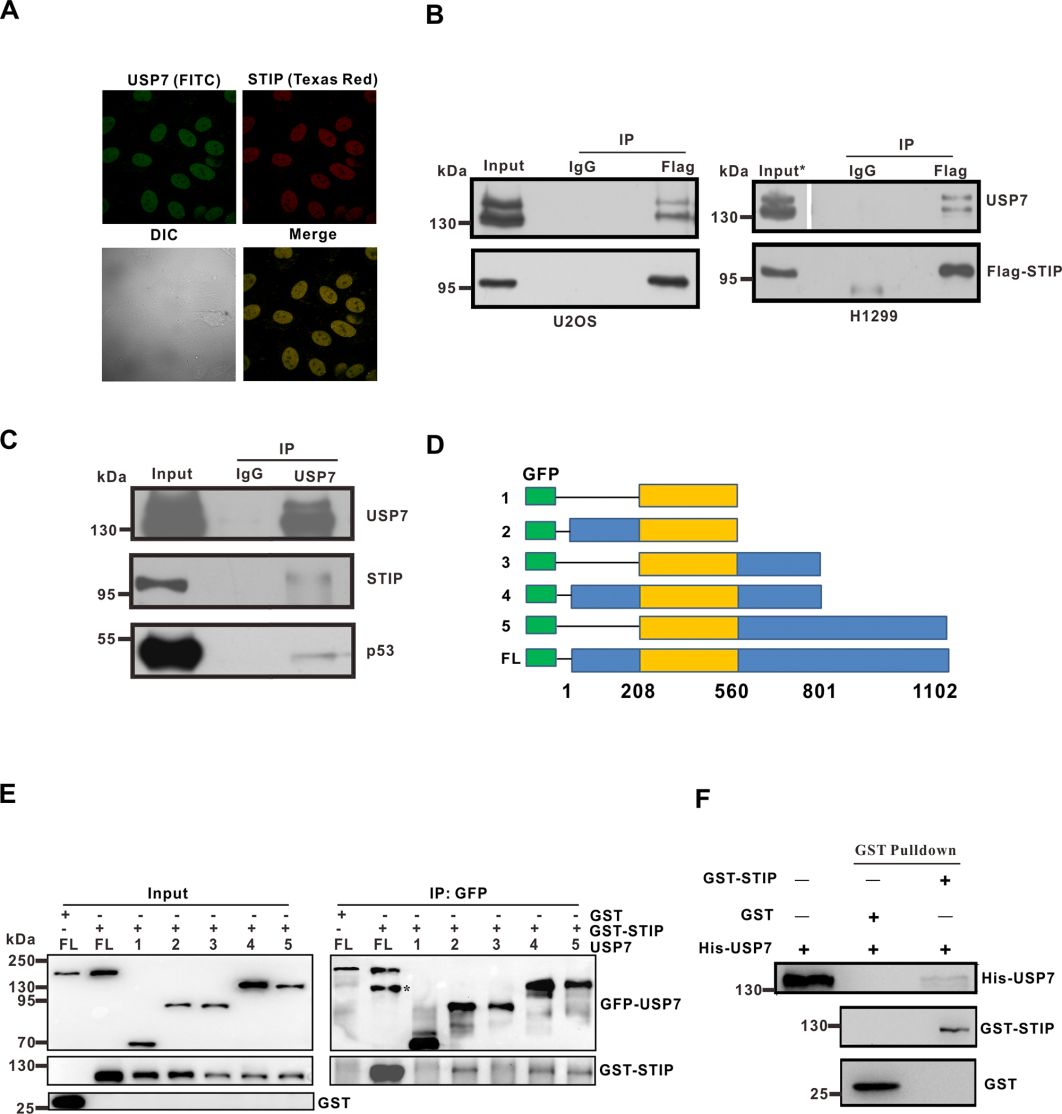
STIP colocalizes and interacts with USP7 **A.** U2OS cells were fixed and incubated with USP7 and STIP antibodies, followed by staining with FITC- or Texas Red-conjugated IgG. USP7 and STIP (upper) and their differential interference contrast (DIC) or merged confocal images (lower) have been shown. **B.** U2OS (p53^+/+^) and H1299 (p53^−/−^) cells were transfected with a Flag-STIP plasmid. Anti-Flag and control IgG antibodies were used for immunoprecipitation, and immunoprecipitates were analyzed by WB using anti-USP7 and anti-Flag antibodies (*To visualize USP7 bands, the exposure time of USP7 in the input is half that of the IP.) **C.** U2OS cell lysates were precipitated with anti-USP7 or control IgG antibodies. The immunoprecipitates were then probed with USP7, STIP, or p53 antibodies. **D.** Schematic representation of plasmids encoding USP7 domains fused to GFP that were used for interaction analyses shown in panel e (1: aa208-560; 2: aa1-560; 3: aa208-801; 4: aa1-801; 5: aa208-1102; FL: full-length). **E.** U2OS cells were transfected with plasmids encoding the indicated GFP-USP7 constructs (defined in panel D), and cell lysates were incubated with GST or GST-STIP and then immunoprecipitated with an anti-GFP antibody. Immunoprecipitates were blotted first with an anti-STIP antibody, and membranes were re-probed with an anti-GFP antibody (*GST-STIP). **F.** USP7 was incubated with GSH-Sepharose beads coupled to GST or GST-STIP. Proteins bound to GSH-Sepharose beads were then eluted and blotted with the indicated antibodies.

A previous study found that GFP-tagged STIP protein formed rod-shaped nuclear polymers termed stiposome [18]. To further explore that subcellular location of endogenous STIP, U2OS cells was labeled with anti-STIP antibody followed by Texas red-conjugated secondary antibody and observed under a confocal microscope. We found that endogenous STIP was localized in nucleoplasm but not nucleoli (Figure 1A; Supplementary Figure S1), confirming previous results obtained from STIP overexpression [18]. Meanwhile, the fluorescent staining was considerably weakened after STIP was knocked down using STIP siRNA, verifying the specificity of anti-STIP antibody (Supplementary Figure 1A).

Using the Eukaryotic Linear Motif Resource (http://elm.eu.org/), we found nine putative USP7-binding domains in STIP. To explore the potential interaction between STIP and USP7 in a cellular context, we first investigated whether endogenous STIP and USP7 colocalize in the nuclear compartment of U2OS cells. Endogenous USP7 and STIP were labeled with their corresponding antibodies conjugated to Texas Red and FITC, respectively. An intense yellow color was observed in those merged images of STIP with USP7, indicating that STIP and USP7 are colocalized in the nucleoplasm (Figure 1A).

To confirm that STIP and USP7 indeed interact *in vivo*, the pCMV-Flag-STIP plasmid was transfected into H1299 (p53^−/−^) and U2OS (p53^+/+^) cells. The expression of Flag-STIP and its interaction with endogenous USP7 were then assessed by coimmunoprecipitation (co-IP) and western blot (WB) analysis. As shown in Figure 1B, USP7 was co-immunoprecipitated in both cell types by an anti-Flag antibody, but not with an isotype-matched negative control IgG. The association of endogenous USP7 and STIP proteins was also investigated by co-IP. As shown, both STIP and p53 (a known interacting partner of USP7) were detected in the anti-USP7 immunoprecipitates from U2OS cells (Figure 1C). Mapping the region of USP7 required for STIP binding revealed that the binding domain for STIP resides in the N-terminal region (amino acids 1-208) and C-terminal region (amino acids 801-1102) (Figure 1D and 1E). To determine whether the STIP-USP7 interaction is direct, we generated recombinant GST-STIP and His-USP7 through *in vitro* translation. Purified GST-STIP interacted with His-USP7 under cell-free conditions (Figure 1F), suggesting a direct interaction occurs between STIP and USP7. These results corroborate the colocalization of STIP and USP7, indicating that STIP interacts directly with USP7 and that their interaction is independent of p53 expression.

### STIP influences the steady-state levels of both Mdm2 and p53, but not USP7

**Figure 2:**
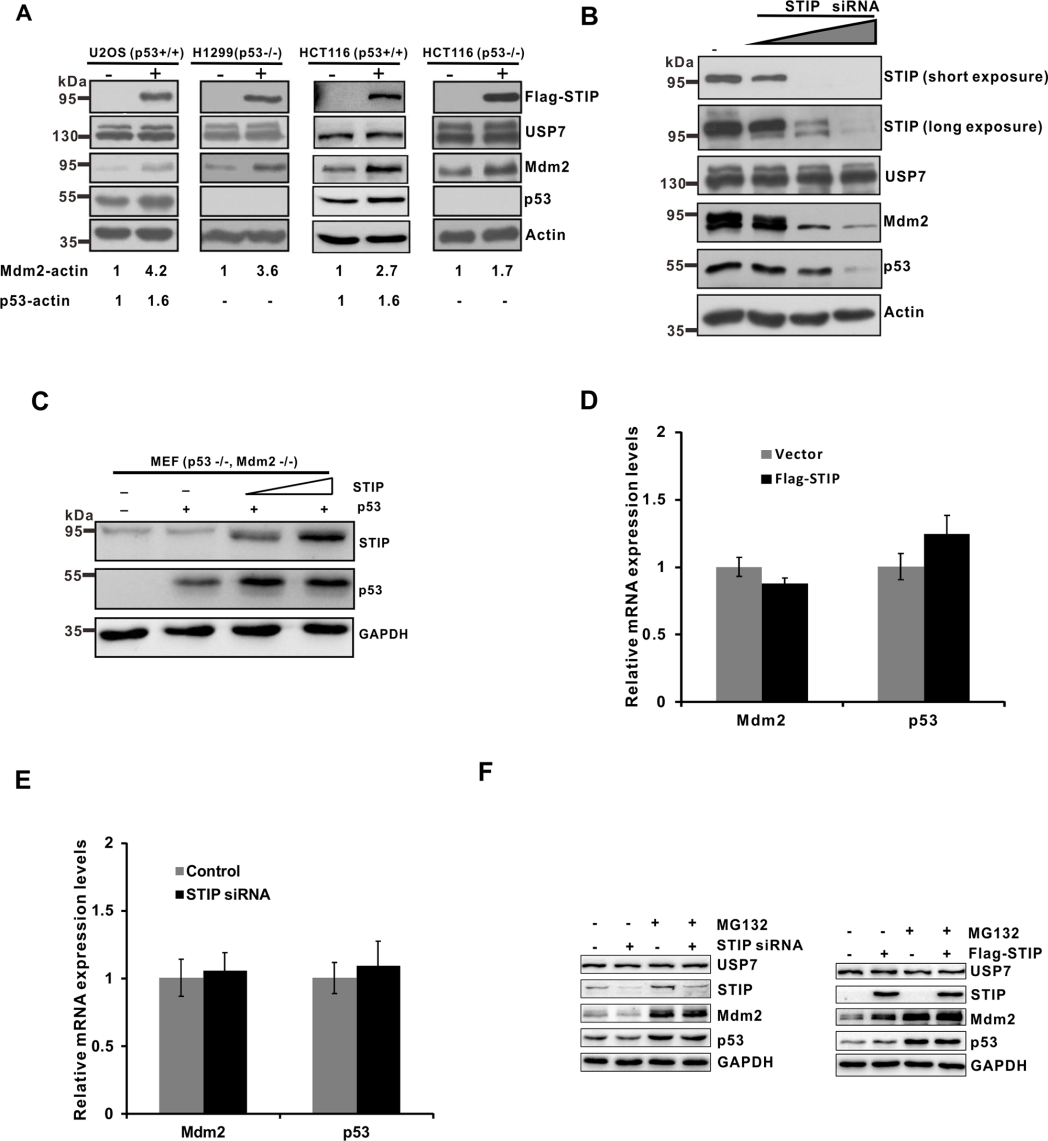
STIP affects the steady-state levels of Mdm2 and p53 **A.** U2OS (p53^+/+^), H1299 (p53^−/−^), HCT116 (p53^+/+^), and HCT116 (p53^−/−^) cells were transfected with a plasmid encoding Flag-STIP (+) or an empty vector control (−). Proteins in lysates were detected by WB with the indicated antibodies. **B.** U2OS cells were transfected with control siRNA and increasing amounts of STIP siRNA. Expression of the indicated proteins was examined by WB with the indicated antibodies. **C.** MEF cells (p53^−/−^, Mdm2^−/−^) were cotransfected with p53 and an increasing amount of the Flag-STIP plasmid. Cell lysates were analyzed by WB with indicated antibodies. **D and E.** U2OS cells were transfected with a plasmid encoding Flag-STIP or an empty vector control (D) or were treated with STIP siRNA or control siRNA (E) for 24 h, and relative mRNA expression levels of Mdm2 and p53 were measured by real-time PCR. **F.** U2OS cells transfected with the Flag-STIP plasmid, control plasmid, STIP siRNA or control siRNA were incubated with 20 μM MG132 for 4 h. Expression of the indicated proteins was examined by WB using the indicated antibodies.

USP7 deubiquitinates Mdm2 and p53, and its overexpression causes Mdm2 and p53 stabilization. [10, 13] Given the interaction of STIP with USP7 identified above, we next investigated whether STIP overexpression affects the steady-state levels of p53, Mdm2, or USP7. STIP overexpression was performed and its effect on the level of three endogenous proteins was assayed in U2OS (p53^+/+^) and H1299 (p53^−/−^), and two HCT116 cell lines with p53 wild-type and null genotypes, respectively. Notably, we observed that STIP overexpression elicited a positive effect on Mdm2, because these four cell lines all manifested a significant increase in the steady-state level of Mdm2 regardless of the status of p53 expression. STIP overexpression also mediated an elevation of the p53 level in cells with wild-type p53, although this effect was more apparent in U2OS than HCT116 cells (Figure 2A). Nonetheless, STIP overexpression did not appear to affect USP7 levels under the same conditions (Figure 2A).

To confirm the role of STIP in regulating Mdm2 and p53 levels, we then knocked down STIP expression in U2OS cells using increasing concentrations of small interfering RNA (siRNA) targeting STIP mRNA. We found that STIP downregulation significantly diminished endogenous levels of Mdm2 and p53 in a dose-dependent manner with no effect on USP7 levels (Figure 2B). Similar effects were observed using STIP-specific short hairpin RNA (shRNA) (Supplementary Figure S2A and S2B). However, the knockdown of STIP did not seem to change the subcellular localization of Mdm2, p53 and USP7 (Supplementary Figure S3). Furthermore, co-transfection of p53 and STIP in MEF cells (p53^−/−^, Mdm2^−/−^) resulted in dose-dependent p53 elevation, indicating that STIP can increase p53 level in an Mdm2-independent manner (Figure 2C). In addition, USP7 overexpression significantly increased endogenous Mdm2 and p53 levels, but had no effect on STIP levels (Supplementary Figure S4A). Moreover, overexpression of Mdm2 or p53 also had no effect on STIP levels (Supplementary Figure S4B and S4C).

To determine whether the effect of STIP on Mdm2 and p53 is mediated at the level of gene transcription, we employed real-time PCR to assess Mdm2 and p53 mRNA expression after overexpression or downregulation of STIP. We found that the levels of Mdm2 and p53 mRNAs were not significantly affected by STIP (Figure 2D and 2E). Hence, the effect of STIP on Mdm2 and p53 steady-state levels is not due to changes in their transcription, but more likely through posttranslational levels. Furthermore, we observed that MG132 treatment stabilized and eventually equalized the changes caused by STIP in Mdm2 and p53 (Figure 2F), suggesting that STIP regulates Mdm2 and p53 levels in a proteasome-dependent manner.

**Figure 3:**
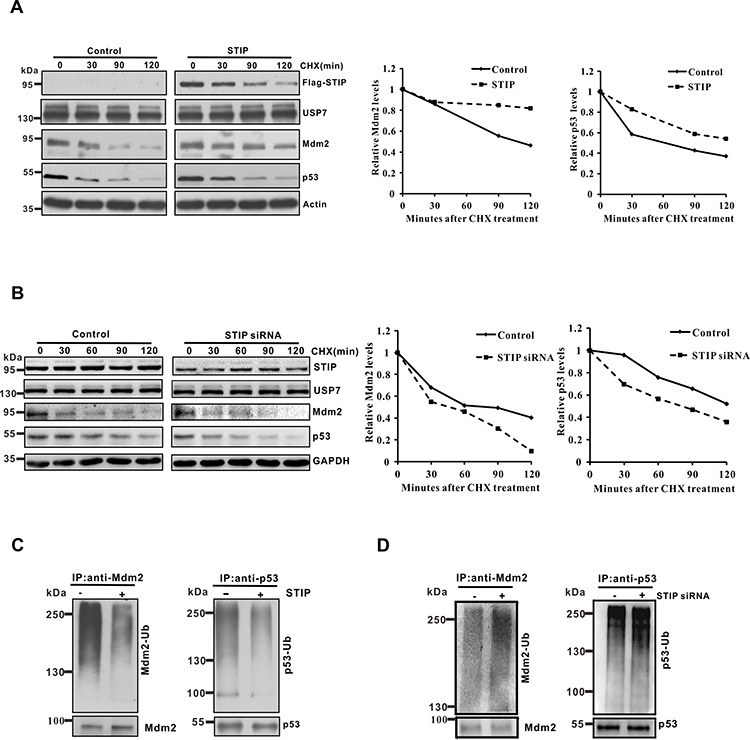
STIP modulates the protein stability of Mdm2 and p53 and their ubiquitination **A and B.** U2OS cells transfected with the Flag-STIP plasmid (A) or STIP siRNA (B) were treated with CHX for indicated time points. To better compare the protein stability of Mdm2 and p53 under different conditions, the blots on the left and their corresponding ones on the right were exposed for different times to achieve similar band intensity at time 0. Right panel: quantification of the Mdm2 and p53 protein levels relative to GAPDH. **C and D.** U2OS cells transfected with the Flag-STIP plasmid (C) or STIP siRNA (D) were incubated with 20 μM MG132 for 4 h. Lysates were immunoprecipitated with anti-Mdm2 or anti-p53 antibodies. The ubiquitination of endogenous Mdm2 and p53 was analyzed by WB using an anti-ubiquitination antibody and either an anti-Mdm2 or an anti-p53 antibody.

To prove that STIP affects Mdm2 and p53 protein stability, cells with or without Flag-STIP were treated with cycloheximide (CHX) to inhibit protein biosynthesis, and protein extracts obtained at different time points were analyzed. As shown in Figure 3A, STIP overexpression increased the protein stability of the Mdm2 and p53 proteins. Conversely, knocking down STIP by siRNA shortened the protein stability of Mdm2 and p53 (Figure 3B). In addition, we found that overexpressed and endogenous STIP have different stability (Figure 3A and 3B). We speculate that unphysiologic overexpression of STIP in U2OS cells may cause feedback regulation of STIP protein levels.

Given that STIP extends the protein stability of Mdm2 and P53, we hypothesized that STIP may be involved in regulating their ubiquitination. To test this hypothesis, we investigated the influence of STIP on the ubiquitination profile of Mdm2 and p53 in U2OS cells. The proteasome inhibitor MG132 was used in Figure 3C and Figure 3D so that polyubiquitinated Mdm2 and p53 proteins would accumulate for increasing the sensitivity of detection. As shown, Mdm2 and p53 ubiquitination was significantly reduced by STIP overexpression (Figure 3C). Conversely, downregulation of STIP increased their ubiquitination (Figure 3D). Similar results were obtained in MCF7 cells (Supplementary Figure S2C). Taken together, these findings provide evidence that STIP critically controls the stability of both Mdm2 and p53 through modulating their ubiquitination and proteasomal degradation.

### STIP is associated with Mdm2 and p53 *in vivo*

**Figure 4:**
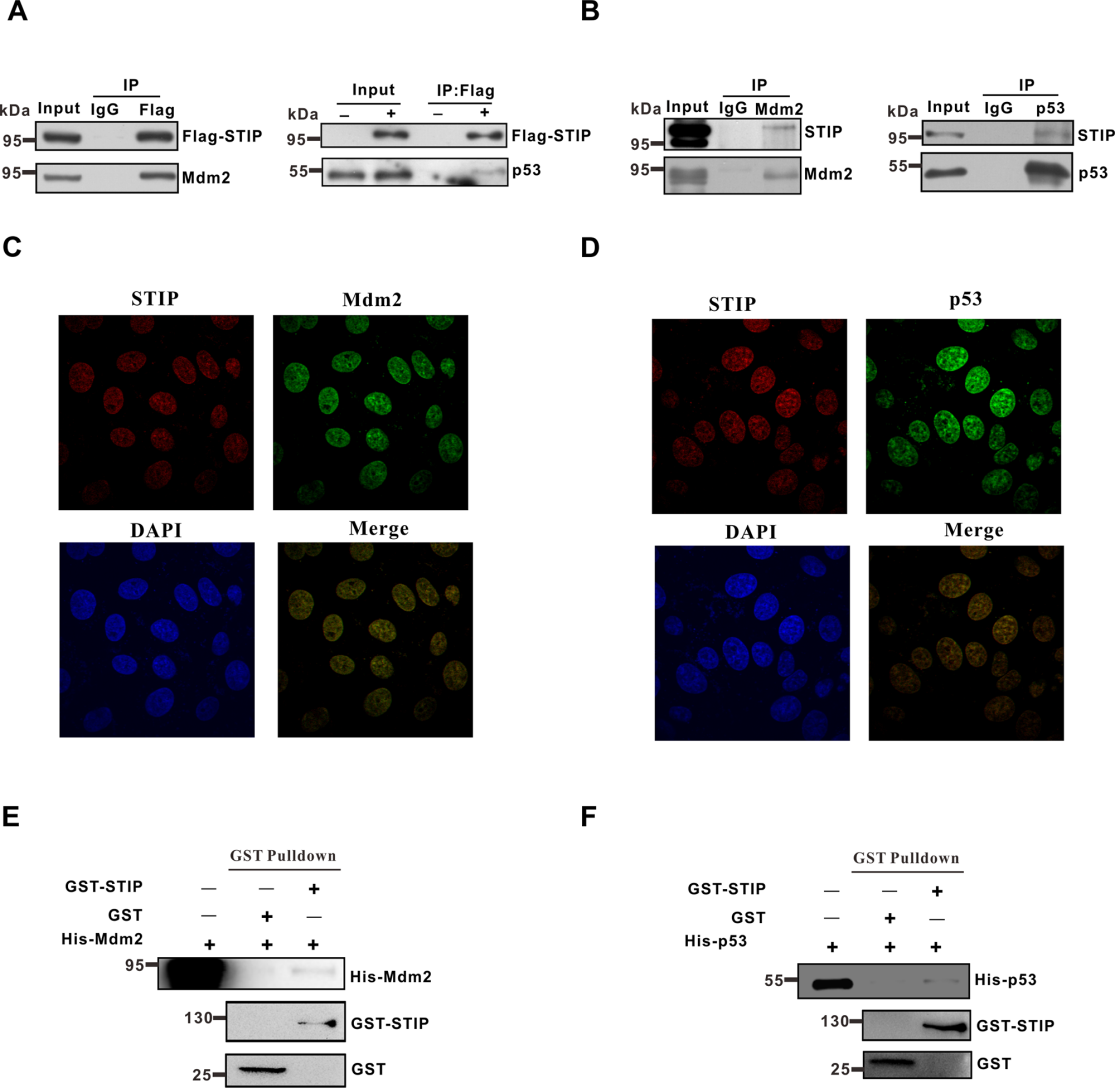
STIP directly interacts with Mdm2 or p53 **A.** U2OS cells were transfected with Flag-STIP or control vectors, and lysates were precipitated with anti-Flag and IgG antibodies. WB analysis of immunoprecipitates and total cell lysates probed with Mdm2 and p53 antibodies are shown. **B.** Endogenous STIP interacts with Mdm2 or p53. Lysates from U2OS cells were precipitated with anti-Mdm2 (left), anti-p53 (right), or control IgG antibodies. Immunoprecipitates and lysates were analyzed by WB with the indicated antibodies. **C and D.** U2OS cells were fixed and incubated with STIP and Mdm2 antibodies (C) or STIP and p53 antibodies (D), followed by staining with FITC- or Texas Red-conjugated IgG. DAPI was used for nuclear counterstaining. **E and F.** The GST and GST-STIP fusion protein were used in a GST pull down assay with *in vitro* translated Mdm2 (E) or p53 (F)

USP7 is known to bind to Mdm2 and p53 via its *N*-terminal TRAF-domain or its C-terminal region in a mutually exclusive manner [10–12]. Because STIP colocalizes and interacts with USP7 *in vivo*, we sought to determine whether STIP also associates with Mdm2 and p53. STIP was overexpressed in U2OS cells and its association with Mdm2 or p53 was assayed by co-IP. As shown, both Mdm2 and p53 were readily co-immunoprecipitated with overexpressed STIP (Figure 4A). To further establish whether the association of STIP with Mdm2 and p53 occurs under natural conditions, reciprocal immunoprecipitation assays of endogenously expressed STIP proteins with anti-Mdm2 and anti-p53 antibodies were performed. The data showed that the immunoprecipitates obtained with both Mdm2 and p53 antibodies retained endogenous STIP (Figure 4B), indicating that STIP associates with both proteins. Meanwhile, immunofluorescence microscope analysis revealed that STIP and Mdm2 or STIP and p53 colocalized in nucleoplasm (Figure 4C and 4D). Furthermore, the STIP-Mdm2 or STIP-p53 interaction is likely to be direct, as shown by an *in vitro* pulldown assay with recombinant protein (Figure 4E and 4F). Taken together, these data provided evidence indicating that STIP may mediate the assembly of multi-component complexes *in vivo*.

### STIP mediates ternary complex assembly as STIP-USP7-Mdm2 or STIP-USP7-p53

**Figure 5:**
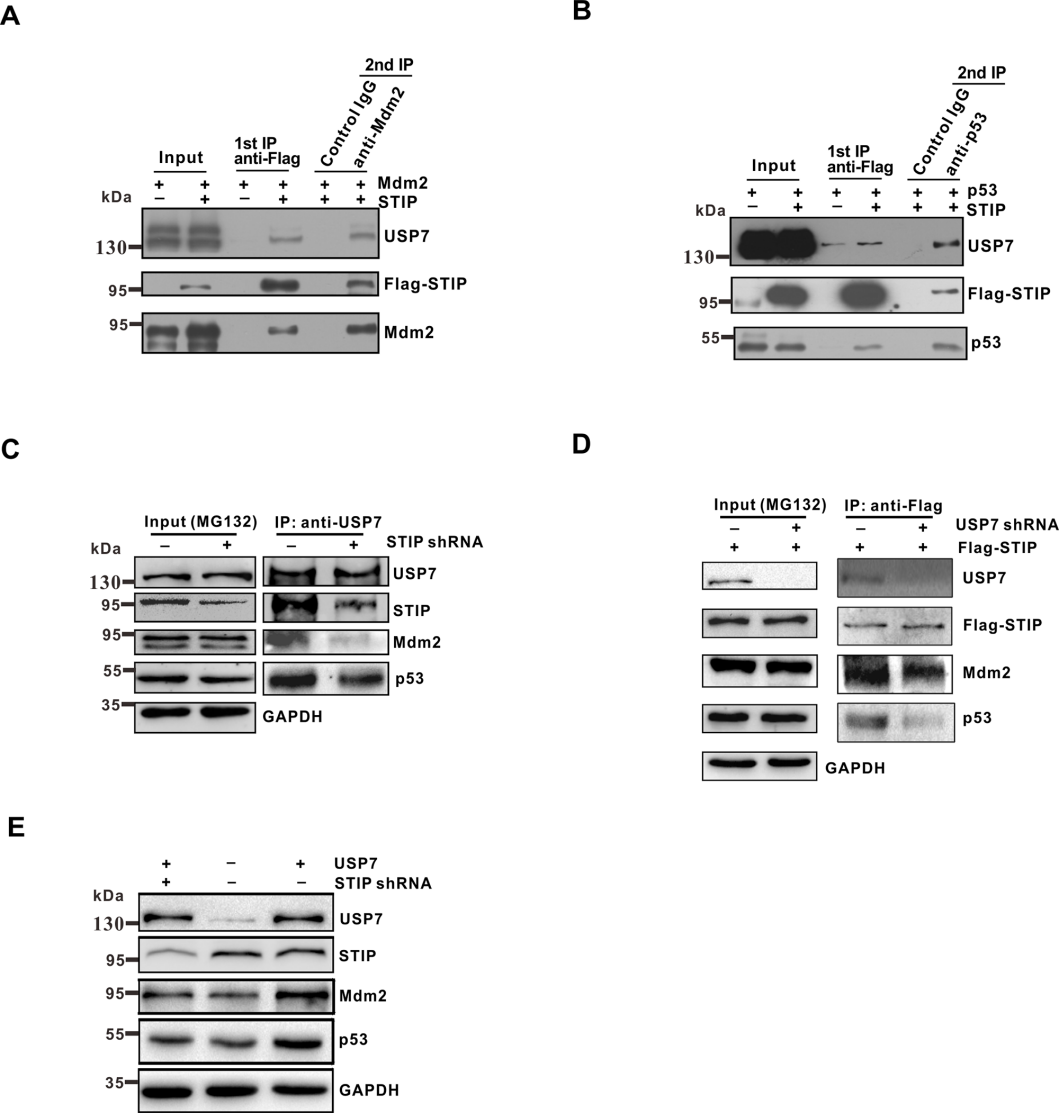
STIP mediates ternary complex assembly as STIP-USP7-Mdm2 or STIP-USP7-p53 **A and B.** U2OS cells were cotransfected overnight with plasmids encoding Flag-STIP plus Mdm2 (A) or Flag-STIP plus p53 (B), followed by MG132 treatment. Cell lysates were precipitated with anti-Flag M2 agarose beads and eluted with Flag peptides. Eluates were further immunoprecipitated with anti-Mdm2, anti-p53, or control IgG antibodies. Protein samples obtained from each step were analyzed by WB with the indicated antibodies. Panel A shows ternary complex STIP-USP7-Mdm2 formation, and panel B shows ternary STIP-USP7-p53 complex formation. **C.** U2OS cells infected with STIP shRNA or control shRNA were incubated with 20 μM MG132 for 4 h, and cell lysates were immunoprecipitated with anti-USP7 antibodies. The indicated proteins were examined by WB. **D.** U2OS cells infected with STIP shRNA or control shRNA were transfected with Flag-STIP plasmid or control plasmid and incubated with 20 μM MG132 for 4 h, and cell lysates were immunoprecipitated with anti-Flag antibodies. The indicated proteins were examined by WB. **E.** U2OS cells treated with STIP shRNA were transfected with USP7. Expression of the indicated proteins were examined by WB.

USP7 can bind directly to Mdm2 or p53 in a mutually exclusive manner. In addition, USP7 can interact indirectly with p53, using Mdm2 as a bridge, resulting in the formation of USP7-Mdm2-p53 complexes. Considering that the effect of STIP on Mdm2 or p53 was independent of each other, we hypothesize that STIP may associate with the USP7-Mdm2 or USP7-p53 complexes. To assess this possibility and define the molecular features of STIP-mediated protein-protein interactions, we transiently coexpressed STIP with either Mdm2 or p53 in U2OS cells. We then employed a two-step immunoprecipitation assay to investigate whether Flag-STIP assembles into different complexes with the other three proteins and, if so, to determine the molecular composition. Following the first immunoprecipitation step with an anti-Flag antibody, we performed a second immunoprecipitation step with anti-Mdm2 or anti-p53 antibodies. We found that endogenous USP7 was present in immunoprecipitates obtained with either anti-Mdm2 (Figure 5A) or anti-p53 (Figure 5B) antibodies. These data indicated that STIP may simultaneously bind to USP7 and either Mdm2 or p53, mediating the assembly of ternary STIP-USP7-Mdm2 and STIP-USP7-p53 complexes, respectively.

The fact that STIP is involved in two different ternary complexes raises the possibility that this interaction may facilitate the USP7-mediated the stabilization of Mdm2 and p53. To test this hypothesis, endogenous STIP was knocked down in U2OS cells. We found that the USP7-Mdm2 or USP7-p53 interactions were significantly reduced in STIP shRNA-treated U2OS cells by co-IP (Figure 5C). Interestingly, STIP knock down seem to have a more profound impact on the interaction between USP7 and Mdm2 than the interaction between USP7 and p53. We speculated that this could be due to USP7 has more extensive interactions with Mdm2 than p53 does under physiological conditions. Meanwhile, co-IP was also performed with anti-Flag antibody after USP7 depletion. We found that the STIP-Mdm2 or STIP-p53 interactions were also significantly reduced (Figure 5D). Furthermore, the effect of USP7 on Mdm2 and p53 was significantly diminished when STIP was knocked down by shRNA (Figure 5E), indicating that STIP plays a significant facilitating role in USP7–mediated stabilization of Mdm2 and P53.

### STIP regulates Mdm2 and p53 levels in a USP7-depenedent manner

**Figure 6:**
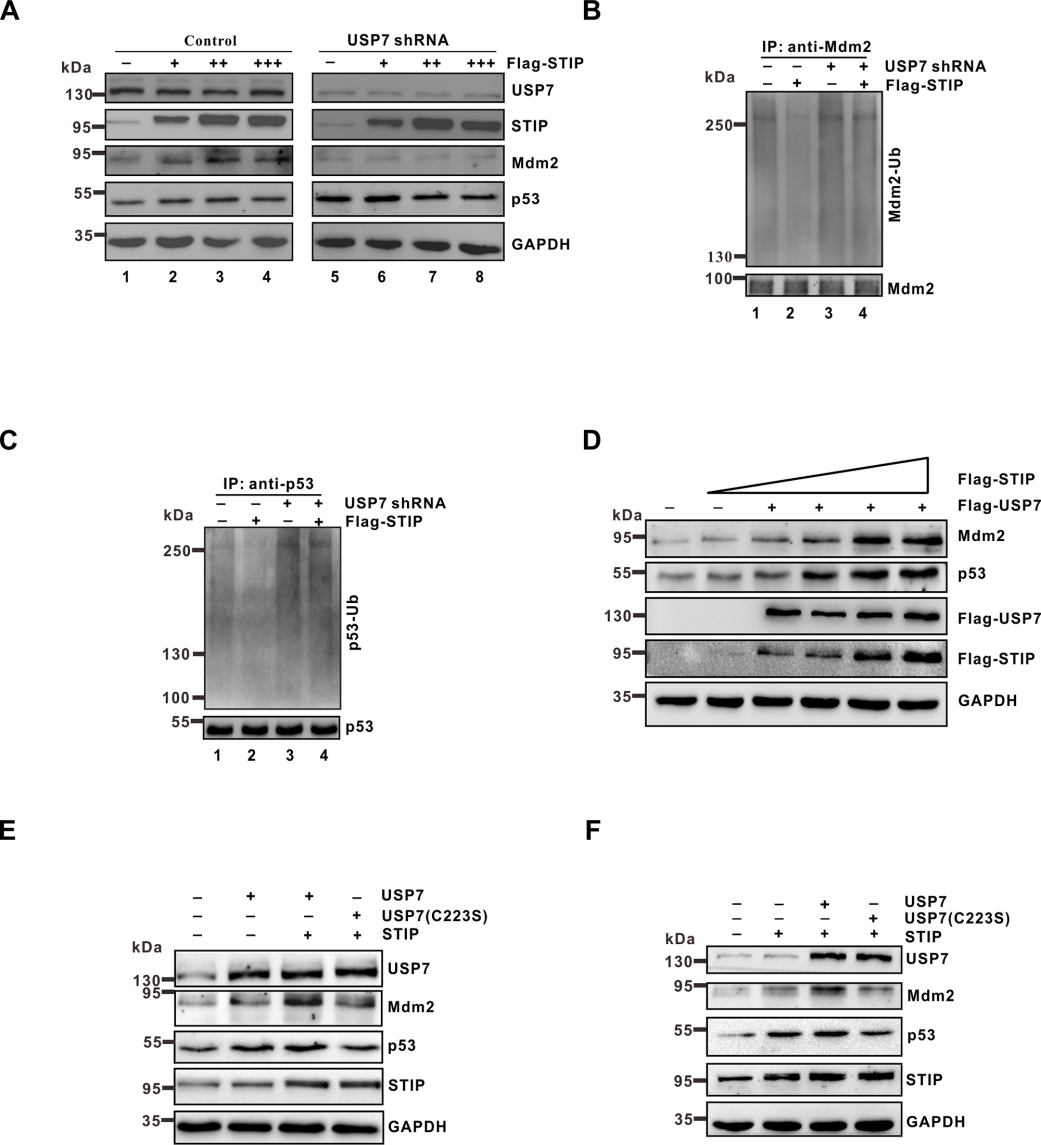
The effect of STIP on Mdm2 and p53 stability depends on USP7 **A.** U2OS cells treated with USP7 shRNA or control shRNA were transfected with increasing amounts of Flag-STIP, and the expression of the indicated proteins was examined by WB. **B and C.** U2OS cells treated with USP7 shRNA or control shRNA were transfected with Flag-STIP or empty vector for 24 h and then incubated with 20 μM MG132 for 4 h. Lysates were immunoprecipitated with anti-Mdm2 (B) or anti-p53 antibodies (C) The ubiquitination of endogenous Mdm2 and p53 was analyzed by WB using an anti-ubiquitination antibody and either an anti-Mdm2 or an anti-p53 antibody. **D.** U2OS cells were cotransfected with the Flag-USP7 plasmid and increasing amounts of the Flag-STIP plasmid, and lysates were immunoblotted for the indicated proteins. **E and F.** U2OS cells were transfected with plasmids encoding USP7, a C223S-USP7 catalytic mutant, or STIP as indicated. Lysates were analyzed by WB using indicated antibodies.

Because STIP and USP7 interact *in vivo* and display similar effects toward Mdm2 or p53, we considered whether STIP regulation of Mdm2 and p53 stability may be mediated through USP7. To investigate this hypothesis, endogenous USP7 was depleted with USP7 shRNA and the effect was compared to control shRNA transfectants in U2OS cells, where STIP was either overexpressed or in the native state. As shown, increasing STIP expression stabilized Mdm2 and p53 in a dose-dependent manner (Figure 6A, lanes 1–4), which was accompanied by a decrease in Mdm2 and p53 ubiquitination (Figure 6B, lanes 1–2; Figure 6C, lanes 1–2). However, when USP7 was knocked down using shRNA, the effect of STIP on Mdm2 and p53 stability and ubiquitination was diminished (Figure 6A, lanes 5–8; Figure 6B, lanes 3–4; Figure 6C, lanes 3–4). Meanwhile, when plasmids expressing STIP and USP7 were cotransfected into U2OS cells, we found that both Mdm2 and p53 were stabilized by STIP in a dose-dependent manner in the presence of USP7 (Figure 6D). In contrast, the effect of STIP on Mdm2 and p53 was significantly diminished in the presence of the catalytic mutant of USP7 (C223S) (Figure 6E and 6F), implying that stabilization of Mdm2 and p53 by STIP depends on USP7 function as a deubiquitinating enzyme. However, STIP itself did not affect the deubiquitinating activity of USP7 (Supplementary Figure S5). Collectively, these findings suggest that STIP acts as a molecular scaffold in concert with USP7 and mediates Mdm2 and p53 stabilization in a UPS7-dependent manner.

### STIP promotes tumor cell growth through USP7

**Figure 7:**
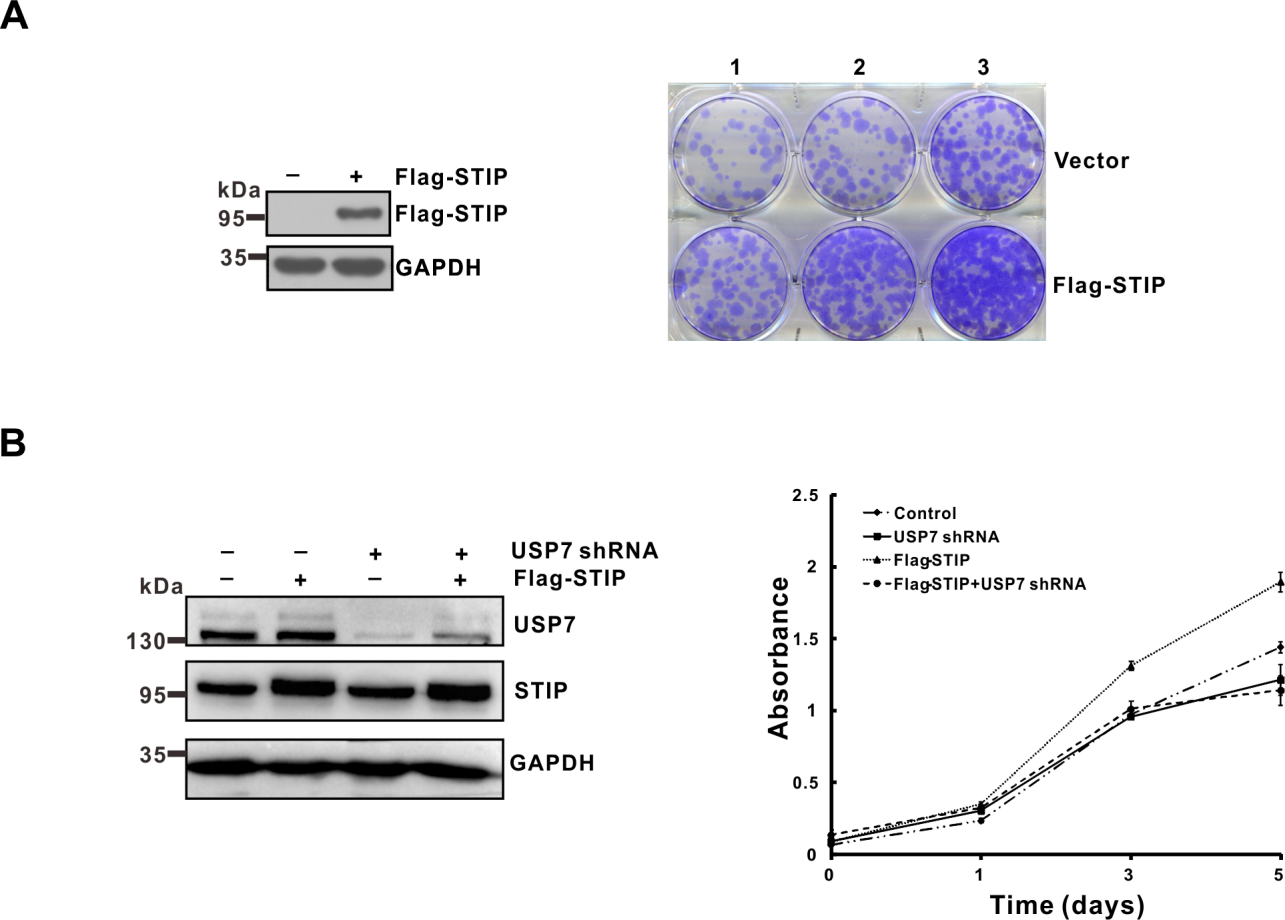
STIP promotes tumor cells growth in a USP7-dependent manner **A.** U2OS cells were transfected with a plasmid encoding Flag-STIP or the parental vector followed by G418 selection. Following a 2-week selection, cells were fixed with methanol and stained with 0.1% crystal violet. Cell seeding density is 1.25 × 10^4^/ml (lane 1), 2.5 × 10^4^/ml (lane 2) and 5 × 10^4^/ml (lane 3) respectively. **B.** U2OS cells infected with lentivirus encoding control or USP7 shRNA were transfected with a plasmid encoding Flag-STIP or the parental vector, and cell growth was monitored by MTT assays performed at the indicated time points.

As shown above, STIP stabilizes Mdm2 and p53, even though the latter two proteins execute opposing functions in various cellular settings [23, 24]. We thus analyzed cell growth to establish the functional significance of STIP in relaying its signal to USP7. U2OS cells were transfected with an empty vector or a plasmid expressing Flag-STIP and cultured for 2 weeks under G418 selection. We found the STIP overexpression increased the number of surviving colonies compared to cells transfected with the control vector (Figure 7A). Conversely, STIP downregulation strongly inhibited cells growth (Figure 7B and Supplementary Figure S2D). Furthermore, downregulation of USP7 effectively attenuated the growth-promoting effect of STIP overexpression (Figure 7B) and the growth-inhibiting effect of STIP downregulation (Supplementary Figure S6A), indicating that cell growth mediated by STIP is dependent on USP7. Given the different affinities of USP7 for Mdm2 and p53 [14, 15], this result suggests that STIP binding may stimulate the USP7-Mdm2 axis more effectively than the USP7-p53 counterpart, thereby facilitating cell growth. In addition, the effect of STIP on cells growth was further evaluated in p53 and Mdm2 depleted MEF cells. Interestingly, we found that either overexpression or downregulation of STIP lost the ability to affect cells growth. This provides evidence that STIP influence cells growth by p53-Mdm2 pathway (Figure S6B and S6C).

## DISCUSSION

Our analysis provides several lines of evidence supporting the conclusion that STIP functions as a molecular scaffolding protein. Firstly, STIP does not appear to possess any intrinsic catalytic activity, in view of the modular arrangement of its conserved domains that are known to engage protein-protein and/or protein-RNA interactions. [18] Secondly, STIP associates with at least two different proteins, namely USP7 and either Mdm2 or p53. Mdm2 and p53 possess catalytic or transcriptional activity, but STIP is itself unaffected by such biological activity. Thirdly, the STIP interaction partners, i.e., USP7 and Mdm2 or p53, bind to each other directly [10, 11, 13, 16], although their binding *in vitro* shows variable affinity [14, 15].

**Figure 8:**
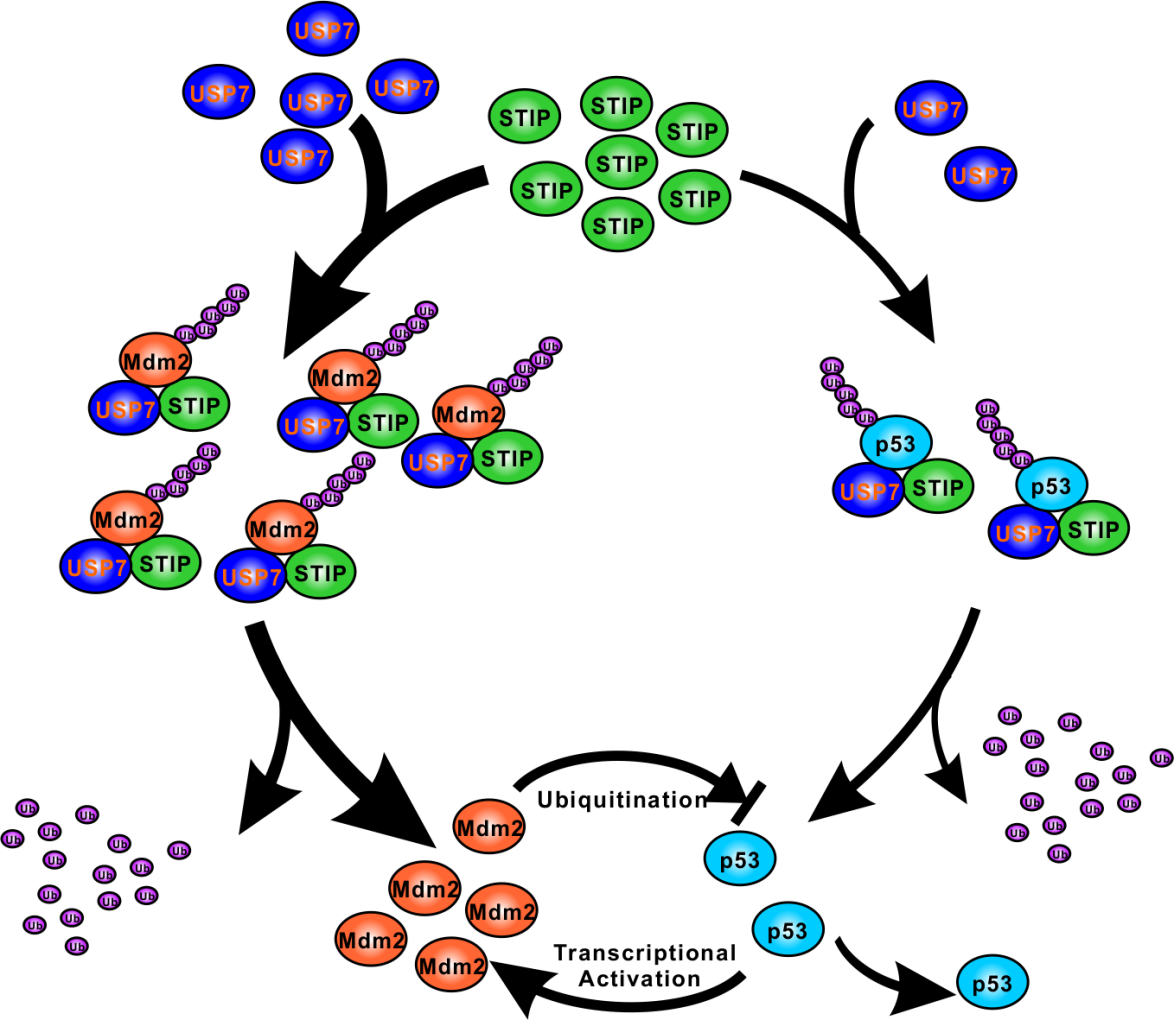
A schematic representation of the role of STIP in regulating Mdm2 and p53 stability In unstressed cells, STIP is simultaneously involved in the assembly of two separate ternary protein complexes as STIP-USP7-Mdm2 and STIP-USP7-p53, which facilitating USP7-mediated stabilization of Mdm2 and p53 by deubiquitination. Since USP7 has more extensive interactions with Mdm2 than p53 does under unstressed cells, STIP stimulates the USP7-Mdm2 axis more effectively than the USP7-p53 counterpart.

Our results demonstrating the interaction between STIP and USP7 and the formation of the ternary complex revealed a novel mechanism whereby USP7 executes its dual-stabilization effect on Mdm2 and p53 via STIP scaffolding. USP7 is a conserved member of the deubiquitinase family involved in cellular stress, epigenetic silencing, cell survival, and viral infection [25–27]. USP7 resides mainly in the nucleus [28], although it also occurs in the cytoplasm and mitochondria [29]. Prior studies indicated that USP7 can be enriched as granules associated with the PML-nuclear body [27]. Here, we show for the first time that a fraction of nuclear USP7 is constitutively associated with STIP. STIP may function as a macromolecular interaction platform to allow deposition and release of active components, thereby regulating their local intensity and positional proximity. We speculate that, from a functional perspective, scaffold organization enables STIP to actively recruit or tether USP7 and its targets Mdm2 and/or p53 from the nucleoplasm, which facilitates USP7-mediated stabilization of Mdm2 and p53 (Figure 8).

USP7 stabilizes p53 and Mdm2 through its deubiquitinase activity and thus plays a key role in regulating p53 and Mdm2 levels under normal and stressed conditions [10, 11, 13, 30]. Indeed, USP7 overexpression increases the steady-state levels of the Mdm2 and p53 protein. However, knockout of USP7 in cells does not decrease p53 levels as predicted, but rather stabilizes p53 [30]. A subsequent study revealed that USP7 exerts different effects on p53 levels between partial reduction and nearly complete ablation of endogenous USP7 [11]. The former induces p53 destabilization, while the latter stabilizes p53 because Mdm2 is degraded in the absence of USP7. Although our results demonstrate that STIP promotes a dual-stabilization effect of USP7 on Mdm2 and p53, it is noteworthy that STIP downregulation does not increase p53 levels. Given that STIP is not known to possess any enzymatic activity and functions as a scaffold, we speculate that STIP knockdown may not completely ablate the function of USP7 as a deubiquitinating enzyme. This notion is supported by the observation that STIP itself did not affect the deubiquitinating activity of USP7. Therefore, STIP knockdown most likely results in direct destabilization of p53, not feedback-mediated p53 stabilization in an Mdm2-dependent manner.

Our results show the effect of STIP on Mdm2 is far stronger than on p53. It is based on the fact that USP7 mainly deubiquitinates and stabilizes Mdm2 in unstressed cells. Consistent with this, our results reveal that STIP knock down have a more profound impact on the interaction between USP7 and Mdm2 than the interaction between USP7 and p53 (Figure 5C). Therefore, STIP may stimulate the USP7-Mdm2 axis more effectively than the USP7-p53 counterpart, thereby facilitating cell growth in spite of functional antagonism between Mdm2 and p53 (Figure 8).

Consistent with the observation that STIP function impinges upon USP7, we identified two ternary complexes, STIP-USP7-Mdm2 and STIP-USP7-p53, which are likely to exist *in vivo*. Notably, a subpopulation of the p53-Mdm2-USP7 ternary complex also occurs *in vivo*, as has been identified [29]. As a scaffold, STIP is likely to function in concert with and depend on USP7 to mediate complex assembly with Mdm2 or p53, enabling USP7 to stabilize both proteins. The dual effect of STIP on p53 and Mdm2 stability is important from a mechanistic point of view, because the STIP-USP7 interaction entails a balancing act in regulating the p53-Mdm2 pathway. Although a multimeric protein complex, such as STIP-USP7-Mdm2-p53, was failed to detected in this study, its dynamic formation in various cellular contexts cannot be excluded and will require further investigation.

## MATERIALS AND METHODS

### Cell culture and transfections

H1299, U2OS and MCF7 cells were cultured in DMEM containing 10% FBS. HCT116 cells were cultured as described [31, 32]. All cells were maintained at 37°C and 5% CO_2_ in a humidified incubator. Transfections with various expression plasmids were performed using HD FuGENE reagents (Roche), according to the manufacturer's suggested protocol.

### Plasmids and antibodies

Full-length STIP was PCR-amplified from human cDNA and subcloned into pCMV-Tag2B (with the Flag tag) to create Flag-tagged STIP expression plasmid, as described [31, 32]. Wild-type pCMV-p53, pCMV-Mdm2, and pENTR-USP7 plasmids (Addgene) have been described previously [29]. Plasmid encoding USP7 domains were generated by PCR and cloned into the pEGFP vector. Anti-p53 (DO-1) (cat. sc-126), anti-Mdm2 (SMP14) (cat. sc-965), anti-fibrillarin (cat. sc-166021) and anti-HAUSP (G-10) (cat. sc-376912) antibodies were purchased from Santa Cruz. Anti-p53 (cat. A300-247A), anti-TFIP11 (cat. A302-548A) and anti-USP7 (cat.A300-033A) antibodies were purchased from Bethyl Laboratories. Anti-Mdm2 (Ab-4) (cat. OP144) was purchased from Millipore. Anti-Flag (M2) (cat. F1804) antibody was purchased from Sigma-Aldrich. Anti-GAPDH (cat. KC-5G4) and anti-actin (cat.KC-5A08) antibodies was purchase from Kangchen Bio-tech. Anti-GFP (cat. 11814460001) antibody was purchased from Roche.

### Western blot analysis

Whole cell lysates were prepared with M-PER buffer containing protease inhibitors (Pierce). Lysates were resolved on 8% SDS-PAGE gels and transferred to nitrocellulose membranes. Membranes were blocked in 5% nonfat milk and incubated with primary antibodies overnight at 4°C. Subsequently, membranes were washed and incubated with appropriate horseradish peroxidase-conjugated secondary antibodies (Santa Cruz). Chemiluminescence was developed using the ECL Kit (Pierce).

### Indirect immunofluorescence

Cells were cultured in Lab-Tek chambers for 24 h, washed three times with PBS, and fixed with 4% paraformaldehyde for 10 min. Fixed cells were washed twice with PBS, permeabilized for 10 min with 0.2% Triton X-100, and blocked for 1 h in 5% BSA before incubation with a primary antibody. Cells were then labeled for 1 h using a FITC- or Texas Red-conjugated secondary antibody (Santa Cruz) and washed three times with PBS. Fluorescence images were acquired using a confocal microscope (Zeiss LSM510).

### Immunoprecipitations

Immunoprecipitations were performed with a Universal Magnetic Co-IP Kit (Active Motif). Briefly, 1 mg of crude extract was incubated overnight at 4°C with 3 μg of a relevant primary antibody or an isotype-matched negative control IgG. Subsequently, samples were incubated for 1 h with 30 μl of magnetic beads conjugated with protein G and then washed four times with Co-IP/wash buffer. Precipitated proteins were dissolved in 2 × SDS sample buffer, boiled, and subjected to WB analysis.

For two-step immunoprecipitations, U2OS cells were cotransfected overnight with pCMV-Flag-STIP and either pCMV-Mdm2 or pCMV-p53 and then treated for 4 h with the proteasome inhibitor MG132 (20 μM). Cells were lysed in IP buffer, passed through a 21-gauge needle, and centrifuged. The resulting supernatants were mixed for 2 h at 4°C with anti-Flag M2-agarose beads. Beads were then centrifuged and washed three times in IP buffer containing 150 mM NaCl. STIP protein complexes were eluted for 2 h at 4°C in 300 μL of IP buffer containing 250 mM NaCl and Flag peptide (300 μg/ml). For the second immunoprecipitation, anti-Mdm2, anti-p53 or control IgG was added to eluates from the first immunoprecipitation. The mixture was kept at 4°C overnight and then incubated with protein G-Sepharose for 2 h at 4°C. Protein samples were subjected to WB analysis using appropriate antibodies.

### RNA interference

Pre-designed STIP siRNA duplexes (sense sequence: 5′-CCUGUUAAGCAGGACGACUtt-3′) and negative control siRNAs were from Ambion. Cells were reverse-transfected with STIP or control siRNAs as specified by the manufacturer. To stably knock down endogenous STIP or USP7 expression in some case, we used lentivirus packing shRNA expression vector (purchased from GenePharma, Shanghai, China) to infect cells. Target cells were infected with lentivirus for 24–48 h according to manufacturer's instruction. STIP shRNA target sequences were: STIP shRNA#1, 5′-TGGGTTGGAAGTCGATGTT-3′; STP shRNA#2, 5′-GTGGATCTTAGATAACATA-3′. USP7 shRNA target sequence was: 5′-AGTCGTTCAGTCGTCGTAT-3′.

### *In vivo* ubiquitination assay

Cells were treated with 20 μM MG132 for 4 h and then lysed in pre-boiled SDS buffer (50 mM Tris-HCl pH 7.5, 0.5 mM EDTA, 1% SDS, 1 mM DTT). Lysates were boiled and cleared by centrifugation. Supernatants were diluted 10-fold in NP-40 buffer (0.5% NP-40, 150 mM NaCl, 50 mM NaF, 50 mM Tris, pH 7.5, 1 mM DTT, 1 mM PMSF, 1 mM Na_3_VO_4_, and protease inhibitors). Following incubation with anti-Mdm2 or anti-p53 antibodies, immunoprecipitates were analyzed by WB using an anti-ubiquitin antibody.

### Protein half-life assays

Cells were treated with cycloheximide (50 μg/ml) for various periods to block protein synthesis. Crude extracts were prepared and protein levels were assessed by WB analysis.

### *In vitro* interaction assay

*In vitro*-translated His-USP7, GST-STIP and GST was prepared in a T7 polymerase-driven wheat germ-coupled transcription and translation system (Cellfree Science). GST pull-down assay was performed by using MagneGST Pull-Down System (Promega). Purified GST-STIP fusion proteins and control GST protein were immobilized on glutathione resins by incubation at 4°C overnight. After beads were washed three times with binding/wash buffer, His-USP7 was applied to immobilized GST fusion protein beads and incubated for 1 h. The beads were washed three times with binding/wash buffer to remove nonspecific binding proteins. The bead-bound proteins were added with SDS sample buffer and boiled for 5 min prior to Western blot analysis.

### Colony formation assay

Cells were transfected with blank pCMV-Tag2B plasmid and pCMV-Tag2B-STIP construct for 48 hours and then plated in six-well plates (U2OS) at 1.25 × 10^4^ cells/ml, 2.5 × 10^4^ cells/ml or 5 × 10^4^ cells/ml, respectively. Cells were cultured in the selection medium (800 μg/ml G418, Sigma). After a 2-week selection, cells were fixed with methanol and stained with 0.1% crystal violet.

## SUPPLEMENTARY FIGURES



## References

[1] Hollstein M, Rice K, Greenblatt MS, Soussi T, Fuchs R, Sorlie T, Hovig E, Smith-Sorensen B, Montesano R, Harris CC (1994). Database of p53 gene somatic mutations in human tumors and cell lines. Nucleic Acids Res.

[2] Vaughan C, Pearsall I, Yeudall A, Deb SP, Deb S (2014). p53: its mutations and their impact on transcription. Sub-cellular biochemistry.

[3] Vogelstein B, Lane D, Levine AJ (2000). Surfing the p53 network. Nature.

[4] Riley T, Sontag E, Chen P, Levine A (2008). Transcriptional control of human p53-regulated genes. Nature reviews Molecular cell biology.

[5] Hock AK, Vousden KH (2014). The role of ubiquitin modification in the regulation of p53. Biochimica et biophysica acta.

[6] Brooks CL, Gu W (2011). p53 regulation by ubiquitin. FEBS letters.

[7] Pflaum J, Schlosser S, Muller M (2014). p53 Family and Cellular Stress Responses in Cancer. Frontiers in oncology.

[8] Fang S, Jensen JP, Ludwig RL, Vousden KH, Weissman AM (2000). Mdm2 is a RING finger-dependent ubiquitin protein ligase for itself and p53. J Biol Chem.

[9] Xu C, Fan CD, Wang X (2015). Regulation of Mdm2 protein stability and the p53 response by NEDD4-1 E3 ligase. Oncogene.

[10] Meulmeester E, Maurice MM, Boutell C, Teunisse AF, Ovaa H, Abraham TE, Dirks RW, Jochemsen AG (2005). Loss of HAUSP-mediated deubiquitination contributes to DNA damage-induced destabilization of Hdmx and Hdm2. Mol Cell.

[11] Li M, Brooks CL, Kon N, Gu W (2004). A dynamic role of HAUSP in the p53-Mdm2 pathway. Mol Cell.

[12] Ma J, Martin JD, Xue Y, Lor LA, Kennedy-Wilson KM, Sinnamon RH, Ho TF, Zhang G, Schwartz B, Tummino PJ, Lai Z (2010). C-terminal region of USP7/HAUSP is critical for deubiquitination activity and contains a second mdm2/p53 binding site. Archives of biochemistry and biophysics.

[13] Li M, Chen D, Shiloh A, Luo J, Nikolaev AY, Qin J, Gu W (2002). Deubiquitination of p53 by HAUSP is an important pathway for p53 stabilization. Nature.

[14] Hu M, Gu L, Li M, Jeffrey PD, Gu W, Shi Y (2006). Structural basis of competitive recognition of p53 and MDM2 by HAUSP/USP7: implications for the regulation of the p53-MDM2 pathway. PLoS Biol.

[15] Sheng Y, Saridakis V, Sarkari F, Duan S, Wu T, Arrowsmith CH, Frappier L (2006). Molecular recognition of p53 and MDM2 by USP7/HAUSP. Nature structural & molecular biology.

[16] Brooks CL, Li M, Hu M, Shi Y, Gu W (2007). The p53—Mdm2—HAUSP complex is involved in p53 stabilization by HAUSP. Oncogene.

[17] Becker K, Marchenko ND, Palacios G, Moll UM (2008). A role of HAUSP in tumor suppression in a human colon carcinoma xenograft model. Cell Cycle.

[18] Ji Q, Huang CH, Peng J, Hashmi S, Ye T, Chen Y (2007). Characterization of STIP, a multi-domain nuclear protein, highly conserved in metazoans, and essential for embryogenesis in Caenorhabditis elegans. Exp Cell Res.

[19] Beausoleil SA, Jedrychowski M, Schwartz D, Elias JE, Villen J, Li J, Cohn MA, Cantley LC, Gygi SP (2004). Large-scale characterization of HeLa cell nuclear phosphoproteins. Proc Natl Acad Sci U S A.

[20] Zhou Z, Licklider LJ, Gygi SP, Reed R (2002). Comprehensive proteomic analysis of the human spliceosome. Nature.

[21] Rappsilber J, Ryder U, Lamond AI, Mann M (2002). Large-scale proteomic analysis of the human spliceosome. Genome Res.

[22] Wen X, Lei YP, Zhou YL, Okamoto CT, Snead ML, Paine ML (2005). Structural organization and cellular localization of tuftelin-interacting protein 11 (TFIP11). Cellular and molecular life sciences : CMLS.

[23] Kruse JP, Gu W (2009). Modes of p53 regulation. Cell.

[24] Alarcon-Vargas D, Ronai Z (2002). p53-Mdm2—the affair that never ends. Carcinogenesis.

[25] van der Horst A, de Vries-Smits AM, Brenkman AB, van Triest MH, van den Broek N, Colland F, Maurice MM, Burgering BM (2006). FOXO4 transcriptional activity is regulated by monoubiquitination and USP7/HAUSP. Nature cell biology.

[26] van der Knaap JA, Kumar BR, Moshkin YM, Langenberg K, Krijgsveld J, Heck AJ, Karch F, Verrijzer CP (2005). GMP synthetase stimulates histone H2B deubiquitylation by the epigenetic silencer USP7. Mol Cell.

[27] Everett RD, Meredith M, Orr A, Cross A, Kathoria M, Parkinson J (1997). A novel ubiquitin-specific protease is dynamically associated with the PML nuclear domain and binds to a herpesvirus regulatory protein. EMBO J.

[28] Fernandez-Montalvan A, Bouwmeester T, Joberty G, Mader R, Mahnke M, Pierrat B, Schlaeppi JM, Worpenberg S, Gerhartz B (2007). Biochemical characterization of USP7 reveals post-translational modification sites and structural requirements for substrate processing and subcellular localization. The FEBS journal.

[29] Marchenko ND, Wolff S, Erster S, Becker K, Moll UM (2007). Monoubiquitylation promotes mitochondrial p53 translocation. EMBO J.

[30] Cummins JM, Rago C, Kohli M, Kinzler KW, Lengauer C, Vogelstein B (2004). Tumour suppression: disruption of HAUSP gene stabilizes p53. Nature.

[31] Baker SJ, Markowitz S, Fearon ER, Willson JK, Vogelstein B (1990). Suppression of human colorectal carcinoma cell growth by wild-type p53. Science.

[32] Oliner JD, Kinzler KW, Meltzer PS, George DL, Vogelstein B (1992). Amplification of a gene encoding a p53-associated protein in human sarcomas. Nature.

