# Supplementary material for: STIP is a critical nuclear scaffolding protein linking USP7 to p53-Mdm2 pathway regulation
Source: Oncotarget. 2015 Oct 10;6(33):34718–31. doi: 10.18632/oncotarget.5303 (PMC4741485; doi:10.18632/oncotarget.5303)
Supplement: Supplementary file 1 [file oncotarget-06-34718-s001.pdf]

## SUPPLEMENTARY FIGURES

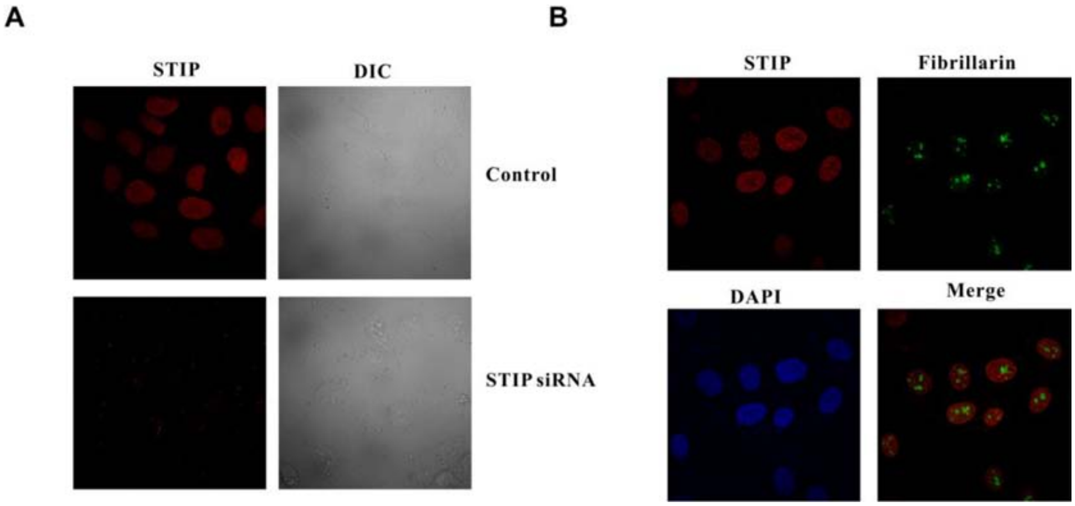


**Supplementary Figure S1: Nuclear localization of endogenous STIP protein. A.** U2OS cells untreated (upper) or treated (lower) with STIP siRNA were fixed and incubated with a primary anti-STIP antibody and a secondary Texas Red-conjugated anti-IgG antibody. **B.** U2OS cells were fixed and incubated with STIP and Fibrillarin antibodies, followed by staining with FITC- or Texas Red-conjugated IgG.

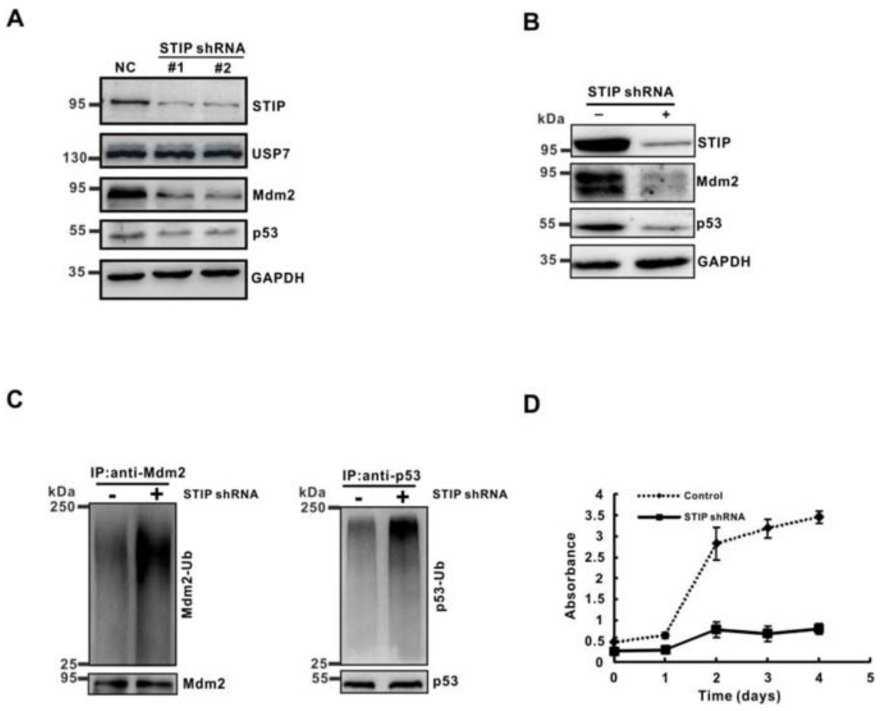


**Supplementary Figure S2: STIP controls the stability of Mdm2 and p53. A.** U2OS cells were infected with lentivirus encoding control shRNA, STIP shRNA #1, or STIP shRNA #2, and lysates were analyzed by WB using indicated antibodies. **B.** MCF7 cells were infected with lentivirus encoding control shRNA or STIP shRNA#2. Expression of the indicated proteins was examined by WB with corresponding antibodies. **C.** MCF7 cells infected with lentivirus encoding STIP shRNA or conrol shRNA were incubated with 20 µM MG132 for 4h. Lysates were immunoprecipitated with anti-Mdm2 or anti-p53 antibodies. Ubiquitination of endogenous Mdm2 and p53 proteins was analyzed by WB using anti-ubiquitin and either anti-Mdm2 or anti-p53 antibodies. **D.** MCF7 cells were infected with lentivirus encoding STIP shRNA, and cell growth was monitored by MTT assays at the indicated time points.

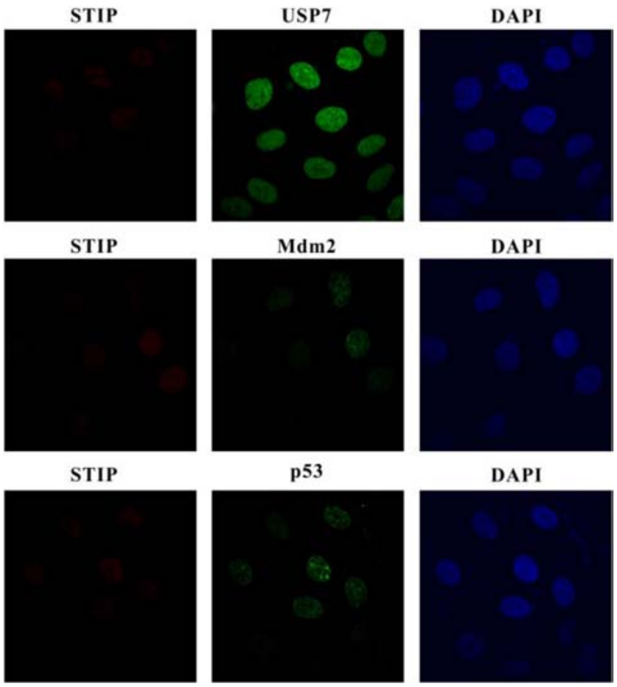


**Supplementary Figure S3: STIP does not affect the subcellular location of USP7, Mdm2 and p53.** After U2OS cells were transfected with STIP siRNA, cells were incubated with indicated antibodies, followed by staining with FITC- or Texas Red-conjugated IgG. DAPI was used for nuclear counterstaining.

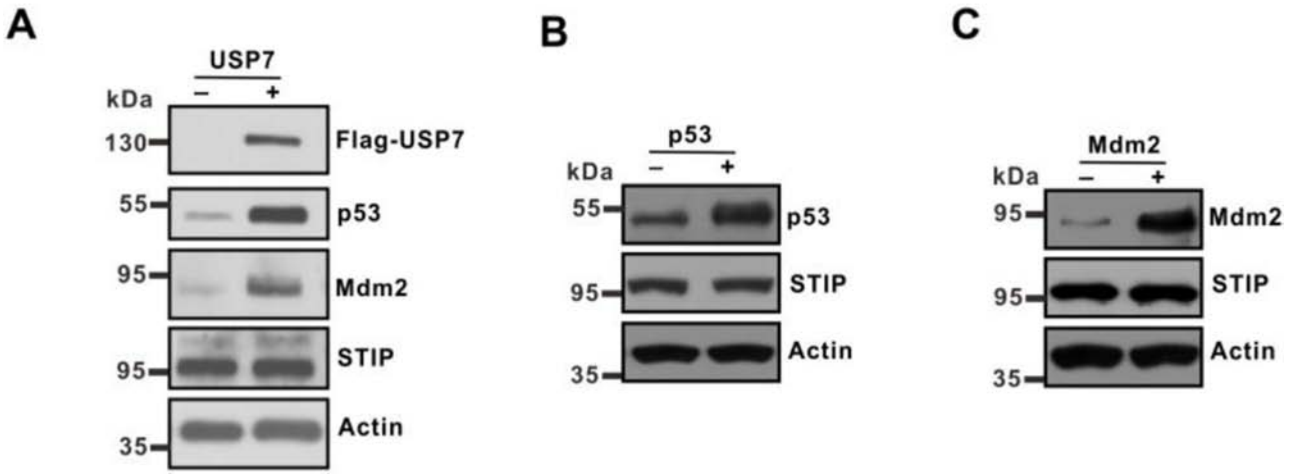


**Supplementary Figure S4: Overexpressed USP7, p53, or Mdm2 does not affect endogenous STIP.** **A, B, C.** U2OS cells were transfected with plasmids encoding USP7 (A), p53 (B), or Mdm2 (C). Lysates were analyzed by WB using the indicated antibodies.

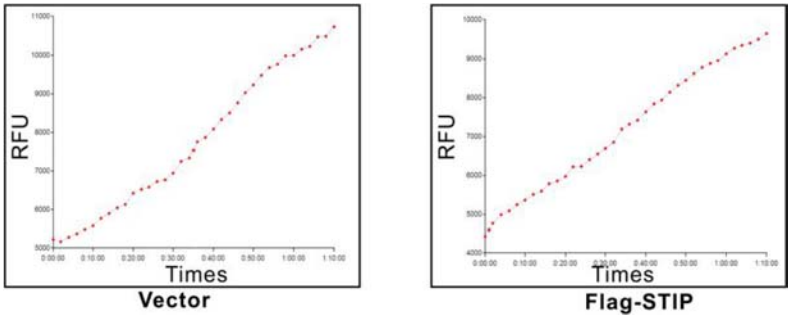


**Supplementary Figure S5: STIP does not affect the deubiquitinating activity of USP7.** U2OS cells were transfected with a control plasmid or a plasmid encoding Flag-STIP. Lysates were immunoprecipitated using an anti-USP7 antibody, and immunoprecipitates were studied in deubiquitination assays using the DUB-Detector Kit from Active Motif.

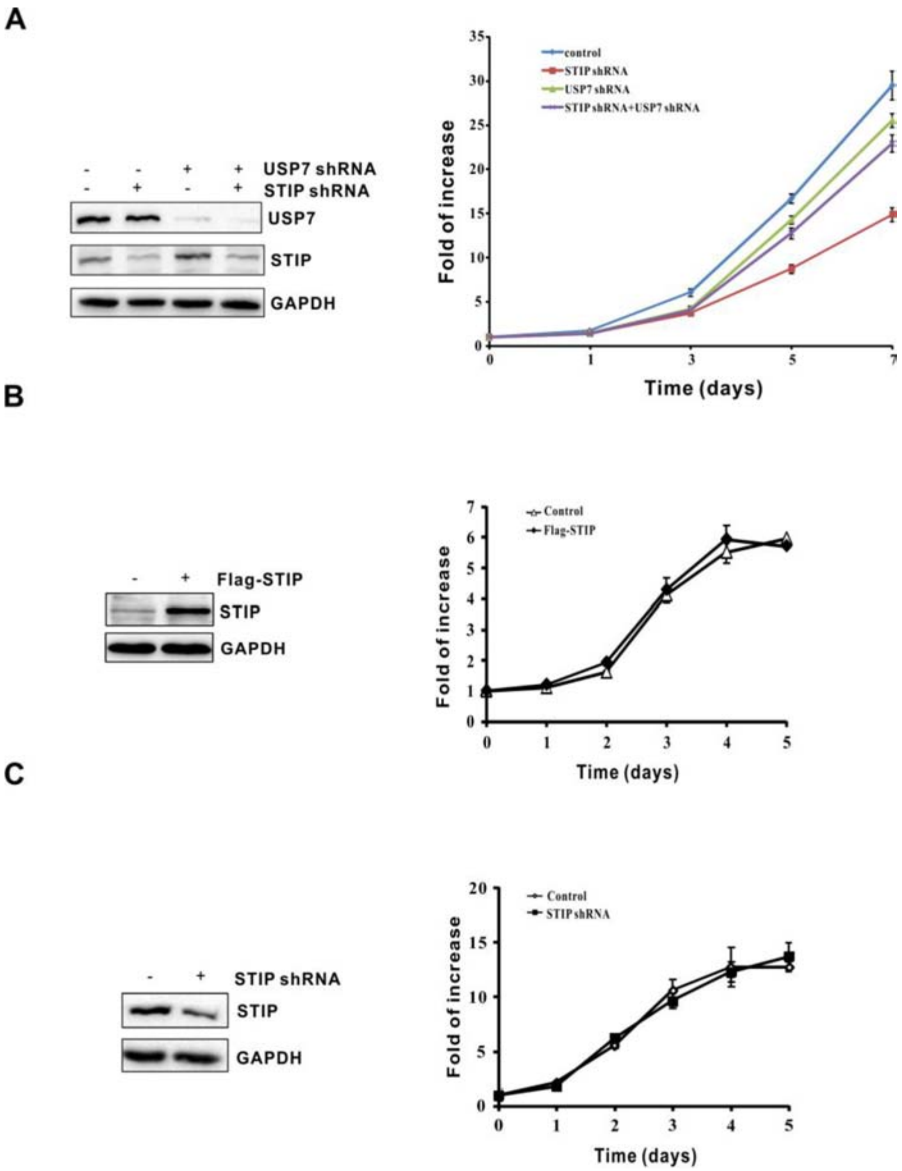


**Supplementary Figure S6: STIP is involved in cells growth. A.** U2OS cells were infected with control, USP7 shRNA or/and STIP shRNA, and cell growth was monitored by MTT assays performed at the indicated time points. **B, C.** Mdm2 and p53 depleted MEF cells were transfected with a plasmid encoding Flag-STIP or the parental vector (B), or were infected with control or STIP shRNA (C), and cell growth was monitored by MTT assays performed at the indicated time points.
